# Supplementary material for: Clinical Significance of Systemic Inflammation Markers in Newly Diagnosed, Previously Untreated Hepatocellular Carcinoma
Source: Cancers (Basel). 2020 May 21;12(5):1300. doi: 10.3390/cancers12051300 (PMC7281027; doi:10.3390/cancers12051300)
Supplement: Supplementary file 1 [file cancers-12-01300-s001.zip › cancers-803067-supp-final-upload/cancers-803067-supp-final.pdf]

Supplementary Materials: Clinical Significance of Systemic Inflammation Markers in Newly Diagnosed, Previously Untreated Hepatocellular Carcinoma

Jeong Il Yu, Hee Chul Park, Gyu Sang Yoo, Seung Woon Paik, Moon Suk Choi, Hye-Seung Kim, Insuk Sohn and Heerim Nam

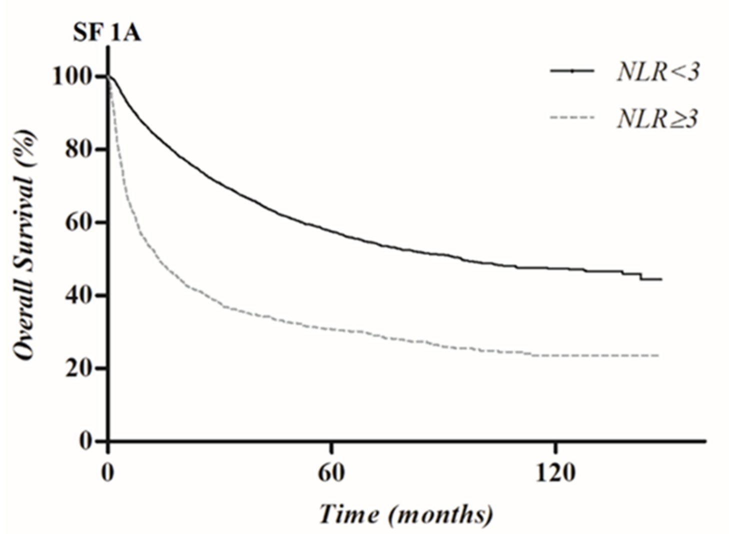

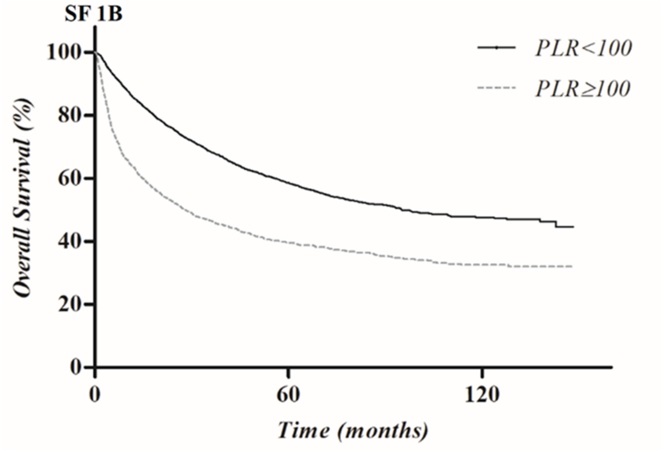

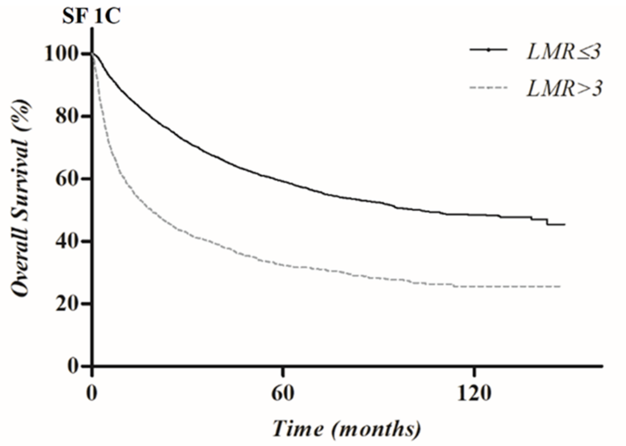

Figure S1. Kaplan-Meier curves of OS according to the systemic inflammation markers (SIMs) as categorical variables in the training set: The survival curves showed clear differences according to the neutrophil to lymphocyte ratio (NLR ≥3, SF 1A), platelet to lymphocyte ratio (PLR ≥100, SF 1B), and lymphocyte to monocyte ratio (LMR ≤ 3, SF 1C).

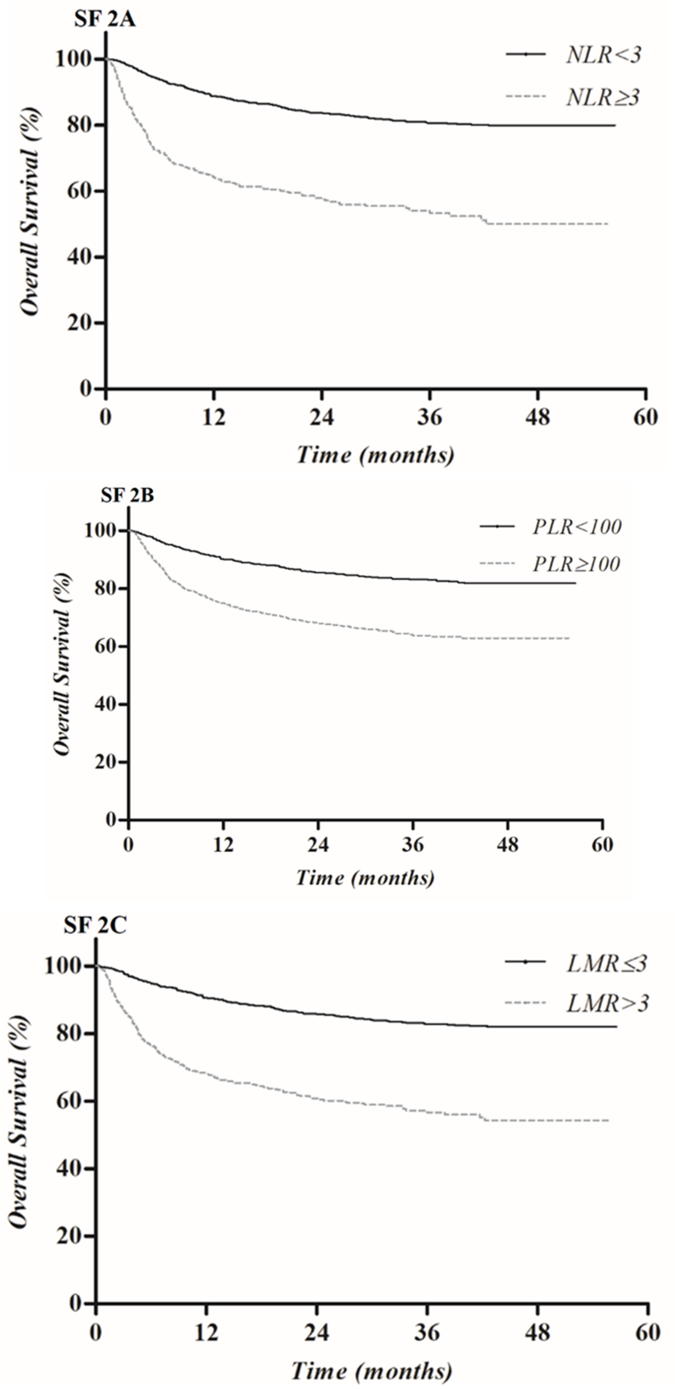

Figure S2. Kaplan-Meier curves of OS according to the SIMs as categorical variables in the validation set: The survival curves showed clear differences according to the NLR (S1A), PLR (S1B), and LMR (S1C).

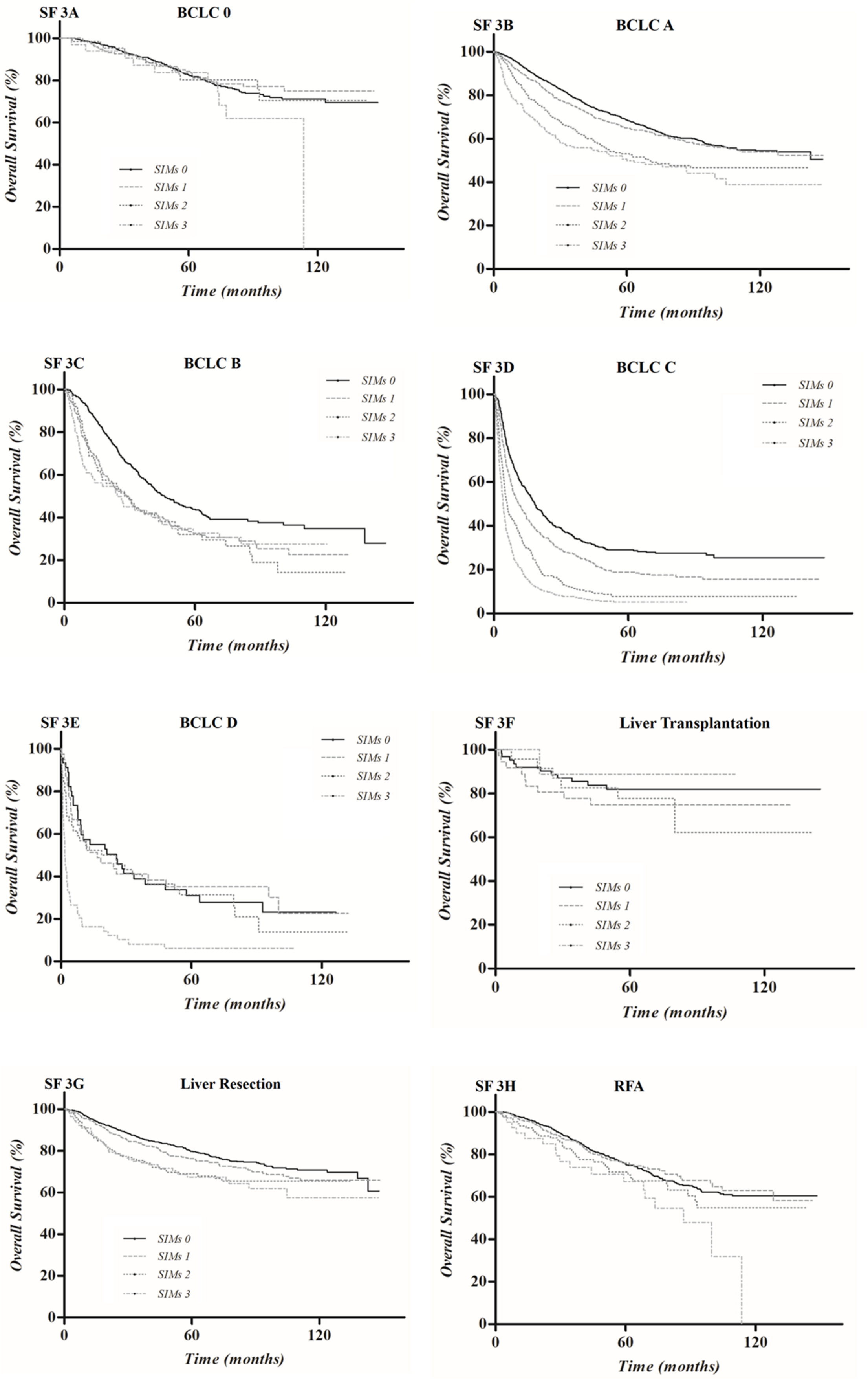
SF 3A
BCLC 0
SIMs 0
SIMs 1
SIMs 2
SIMs 3
Overall Survival (%)
Time (months)
SF 3B
BCLC A
SIMs 0
SIMs 1
SIMs 2
SIMs 3
Overall Survival (%)
Time (months)
SF 3C
BCLC B
SIMs 0
SIMs 1
SIMs 2
SIMs 3
Overall Survival (%)
Time (months)
SF 3D
BCLC C
SIMs 0
SIMs 1
SIMs 2
SIMs 3
Overall Survival (%)
Time (months)
SF 3E
BCLC D
SIMs 0
SIMs 1
SIMs 2
SIMs 3
Overall Survival (%)
Time (months)
SF 3F
Liver Transplantation
SIMs 0
SIMs 1
SIMs 2
SIMs 3
Overall Survival (%)
Time (months)
SF 3G
Liver Resection
SIMs 0
SIMs 1
SIMs 2
SIMs 3
Overall Survival (%)
Time (months)
SF 3H
RFA
Overall Survival (%)
Time (months)

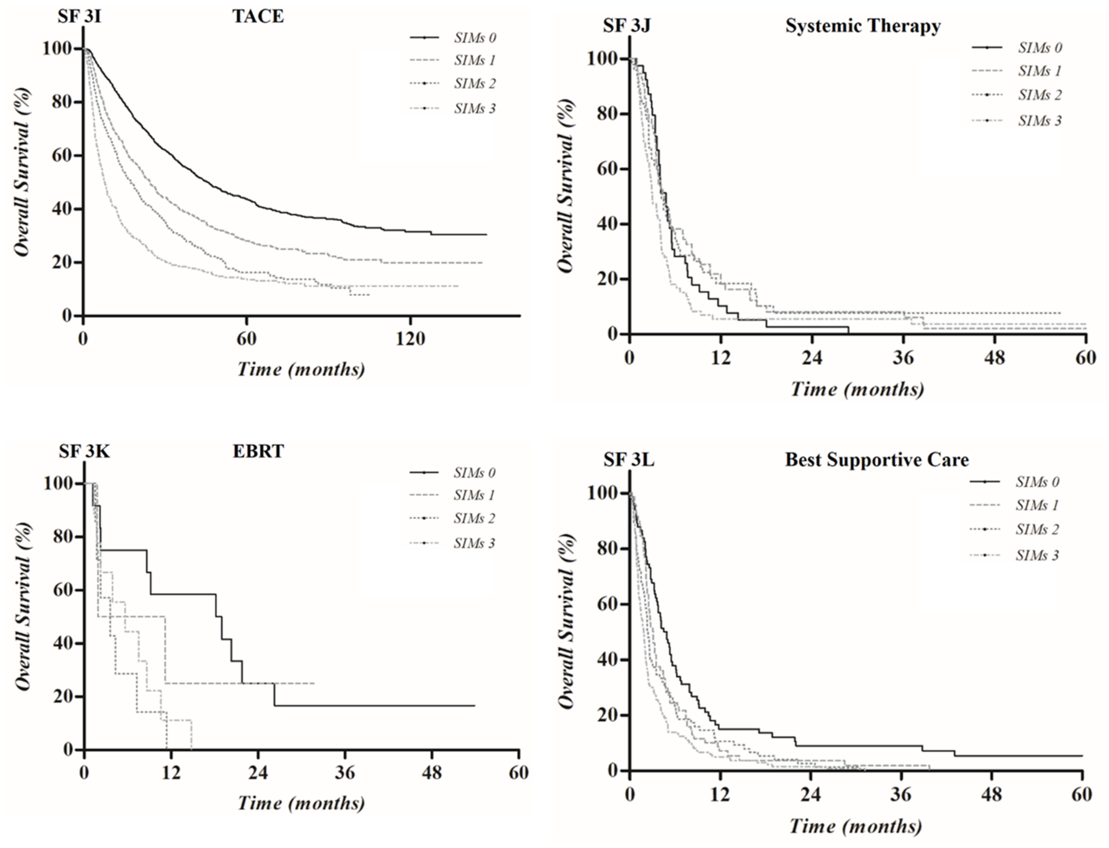

Figure S3. Kaplan-Meier curves of OS according to the SIMs and BCLC staging systems or treatment modalities in the training set.

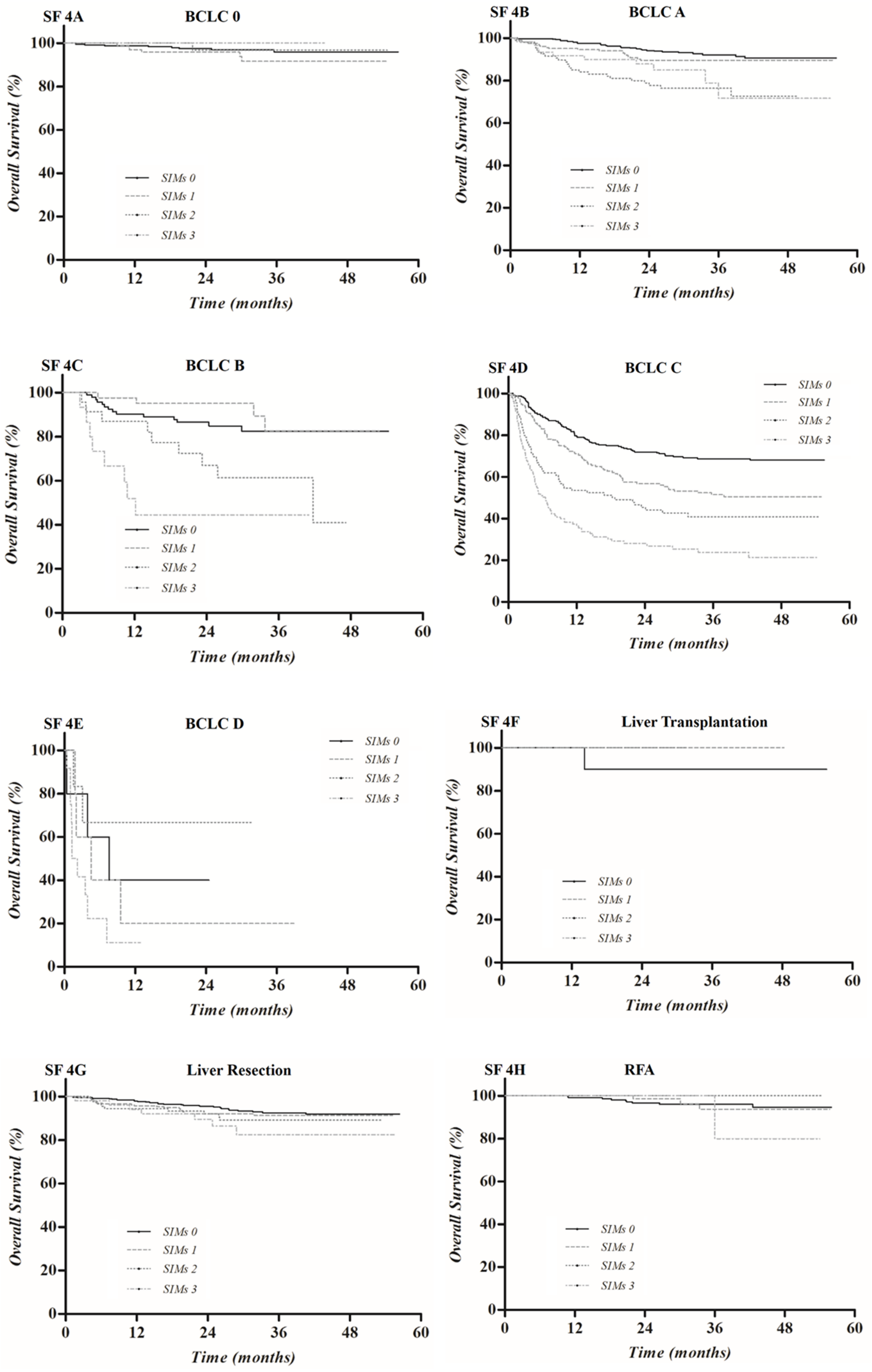
SF 4A
BCLC 0
SF 4B
BCLC A
SF 4C
BCLC B
SF 4D
BCLC C
SF 4E
BCLC D
SF 4F
Liver Transplantation
SF 4G
Liver Resection
SF 4H
RFA
Overall Survival (%)
Time (months)
SIMs 0
SIMs 1
SIMs 2
SIMs 3
0
20
40
60
80
100
0
12
24
36
48
60

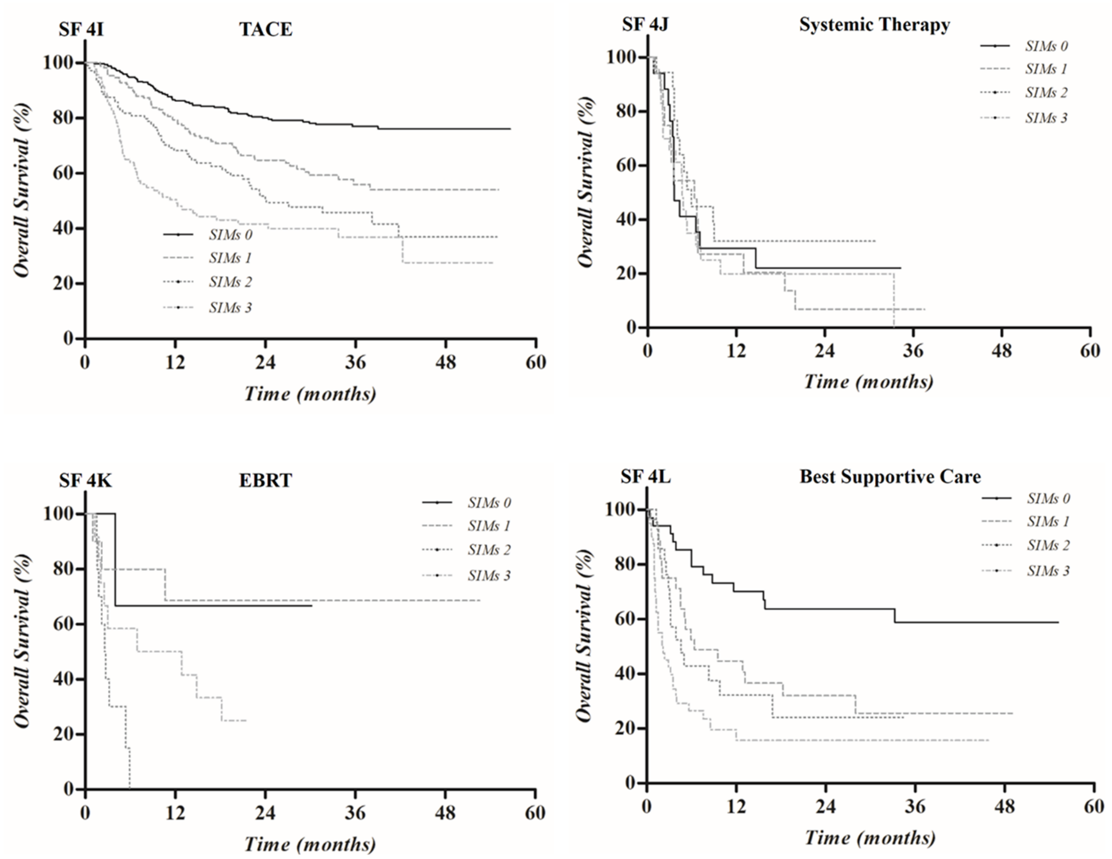

Figure S4. Kaplan-Meier curves of OS according to the SIMs and BCLC staging systems or treatment modalities in the validation set.

Table S2. Correlation Analysis between Neutrophil to Lymphocyte Ratio (NLR) as the Categorical Variable and Other Variables in the Training Set.

**Variables**		**NLR < 3 (*n* = 5218)**	**NLR ≥ 3 (*n* = 1361)**	***p*-Value**
**Age (years)**	Median	57	56	0.001
	Range	13–87	16-88	
Sex	Male	4142 (79.4)	1113 (81.8)	0.053
	Female	1075 (20.6)	248 (18.2)	
ECOG performance status	0	4852 (93.0)	1132 (83.2)	<0.001
	1	302 (5.8)	162 (11.9)	
	2	37 (0.7)	25 (1.8)	
	3	19 (0.4)	26 (1.9)	
	4	8 (0.2)	16 (1.2)	
Cause of hepatitis	HBV	3878 (74.3)	1063 (78.1)	0.053
	HCV	514 (9.9)	124 (9.1)	
	HBV/HCV	47 (0.9)	11 (0.8)	
	Alcohol	235 (4.5)	50 (3.7)	
	Unknown	544 (10.4)	113 (8.3)	
Child-Pugh Class	A	4569 (87.6)	1000 (73.5)	<0.001
	B	592 (11.3)	297 (21.8)	
	C	57 (1.1)	64 (4.7)	
Glasgow prognostic scale*	0	1181 (41.6)	207 (23.4)	<0.001
	1	1391 (49.0)	483 (54.5)	
	2	268 (9.4)	196 (22.1)	
BCLC stage	0	918 (17.6)	94 (6.9)	<0.001
	A	2555 (49.0)	447 (32.8)	
	B	646 (12.4)	112 (8.2)	
	C	1017 (19.5)	613 (45.0)	
	D	82 (1.6)	95 (7.0)	
ALBI grade	I	2942 (56.4)	549 (40.3)	<0.001
	II	2078 (39.8)	690 (50.7)	
	III	198 (3.8)	122 (9.0)	
Portal vein invasion	Vp0	4575 (87.7)	884 (65.0)	<0.001
	Vp1	274 (5.3)	154 (11.3)	
	Vp2	102 (2.0)	77 (5.7)	
	Vp3	75 (1.4)	48 (3.5)	
	Vp4	192 (3.7)	198 (14.5)	
T stage	1	1072 (20.5)	136 (11.3)	<0.001
	2	2420 (46.4)	484 (35.6)	
	3	1422 (27.3)	518 (38.1)	
	4	304 (5.8)	223 (16.4)	
N stage	0	4991 (95.6)	1166 (85.7)	<0.001
	1	227 (4.4)	195 (14.3)	
M stage	0	5074 (97.2)	1200 (88.2)	<0.001
	1	144 (2.8)	161 (11.8)	
AFP (ng/mL)	Median	30	185	<0.001
	Range	1–600000	1–600000	
PIVKA-II (mAU/mL)	Median	54	500	<0.001
	Range	2–75000	5–75000	
Primary treatment	Liver transplantation	96 (1.8)	32 (2.4)	<0.001
	Hepatectomy	1599 (30.6)	270 (19.8)	
	Radiofrequency ablation	1172 (22.5)	146 (10.7)	
	TACE	2045 (39.2)	570 (41.9)	
	Systemic therapy	118 (2.3)	134 (9.8)	
	Radiotherapy	18 (0.3)	14 (1.0)	
	None	170 (3.3)	195 (14.3)	
PLR	Median	74.5	133.2	<0.001
	Range	2.8–793.7	24.4–638.8	
LMR	Median	4.5	2.2	<0.001
	Range	0.7–92.0	0.3–30.3	

Abbreviation: NLR = neutrophil to lymphocyte ratio; ECOG = Eastern Cooperative Oncology Group; HBV = hepatitis B virus; HCV = hepatitis C virus; BCLC = Barcelona Clinic Liver Cancer; ALBI = albumin-bilirubin; AFP = alpha-fetoprotein; PIVKA-II = Protein induced by vitamin K absence or

antagonist-**II**; TACE = trans-arterial chemo-embolization; PLR = platelet to lymphocyte ratio; LMR = lymphocyte to monocyte ratio. Glasgow prognostic scale*: The C-reactive protein (CRP) test was performed selectively in some patients.

Table S3. Correlation Analysis between Platelet to Lymphocyte Ratio (PLR) as the Categorical Variable and Other Variables in the Training Set.

Variables		PLR < 100 (*n* = 4293)	PLR ≥ 100 (*n* = 2285)	*p*-Value
**Age (years)**	Median	57	56	<0.001
	Range	27–87	13–88	
Sex	Male	3438 (80.1)	1817 (79.5)	0.583
	Female	855 (19.9)	468 (20.5)	
ECOG performance status	0	4011 (93.4)	1973 (86.3)	<0.001
	1	219 (5.1)	245 (10.7)	
	2	31 (0.7)	31 (1.4)	
	3	18 (0.4)	27 (1.2)	
	4	15 (0.3)	9 (0.4)	
Cause of hepatitis	HBV	3207 (74.7)	1734 (75.9)	0.115
	HCV	402 (9.4)	236 (10.3)	
	HBV/HCV	43 (1.0)	15 (0.7)	
	Alcohol	191 (4.4)	94 (4.1)	
	Unknown	451 (10.5)	206 (9.0)	
Child-Pugh Class	A	3638 (84.7)	1931 (84.5)	0.863
	B	575 (13.4)	314 (13.7)	
	C	81 (1.9)	40 (1.8)	
Glasgow prognostic scale*	0	928 (41.8)	460 (30.5)	<0.001
	1	1064 (47.9)	810 (53.8)	
	2	228 (10.3)	236 (15.7)	
BCLC stage	0	813 (18.9)	199 (8.7)	<0.001
	A	2088 (48.6)	914 (40.0)	
	B	538 (12.5)	220 (9.6)	
	C	745 (17.3)	885 (38.7)	
	D	110 (2.6)	67 (2.9)	
ALBI grade	I	2231 (52.0)	1260 (55.1)	0.028
	II	1841 (42.9)	927 (40.6)	
	III	222 (5.2)	98 (4.3)	
Portal vein invasion	Vp0	3824 (89.1)	1635 (71.6)	<0.001
	Vp1	186 (4.3)	242 (10.6)	
	Vp2	79 (1.8)	100 (4.4)	
	Vp3	55 (1.3)	68 (3.0)	
	Vp4	150 (3.5)	240 (10.5)	
T stage	1	980 (22.8)	228 (10.0)	<0.001
	2	1941 (45.2)	963 (42.1)	
	3	1132 (26.4)	808 (35.4)	
	4	241 (5.6)	286 (12.5)	
N stage	0	4111 (95.7)	2046 (89.5)	<0.001
	1	183 (4.3)	239 (10.5)	
M stage	0	4189 (97.6)	2085 (91.2)	<0.001
	1	105 (2.4)	200 (8.8)	
AFP (ng/mL)	Median	26	128	<0.001
	Range	1-200000	1–600000	
PIVKA-II (mAU/mL)	Median	34	336	<0.001
	Range	2–75000	3–75000	
Primary treatment	Liver transplantation	111 (2.6)	17 (0.7)	<0.001
	Hepatectomy	1123 (26.2)	746 (32.6)	
	Radiofrequency ablation	1083 (25.2)	235 (10.3)	
	TACE	1731 (40.3)	884 (38.7)	
	Systemic therapy	87 (2.0)	165 (7.2)	
	Radiotherapy	16 (0.4)	16 (0.7)	
	None	143 (3.3)	222 (9.7)	
NLR	Median	1.5	2.7	<0.001
	Range	0.1–18.7	0.2–47.8	
LMR	Median	4.6	3.0	<0.001
	Range	0.3–92.0	0.4–30.3	

Abbreviation: PLR = platelet to lymphocyte ratio; ECOG = Eastern Cooperative Oncology Group; HBV = hepatitis B virus; HCV = hepatitis C virus; BCLC = Barcelona Clinic Liver Cancer; ALBI = albumin-bilirubin; AFP = alpha-fetoprotein; PIVKA-II = Protein induced by vitamin K absence or antagonist-**II**; TACE = trans-arterial chemo-embolization; NLR = neutrophil to lymphocyte ratio; LMR = lymphocyte to monocyte ratio. Glasgow prognostic scale*: The C-reactive protein (CRP) test was performed selectively in some patients.

Table S4. Correlation Analysis between Lymphocyte to Monocyte Ratio (LMR) as the Categorical Variable and Other Variables in the Training Set.

**Variables**		**LMR < 3 (*n* = 1775)**	**LMR ≥ 3 (*n* = 4803)**	***p*-Value**
**Age (years)**	Median	57	56	0.306
	Range	27–87	13-88	
Sex	Male	1484 (83.6)	3771 (78.5)	<0.001
	Female	291 (16.4)	1032 (21.5)	
ECOG performance status	0	1512 (85.2)	4472 (93.1)	<0.001
	1	193 (10.9)	271 (5.6)	
	2	30 (1.7)	32 (0.7)	
	3	25 (1.4)	20 (0.4)	
	4	15 (0.8)	9 (0.2)	
Cause of hepatitis	HBV	1362 (76.7)	3579 (74.5)	0.401
	HCV	166 (9.4)	472 (9.8)	
	HBV/HCV	15 (0.8)	43 (0.9)	
	Alcohol	73 (4.1)	212 (4.4)	
	Unknown	159 (9.0)	498 (10.4)	
Child-Pugh Class	A	1258 (70.9)	4311 (89.7)	<0.001
	B	434 (24.5)	455 (9.5)	
	C	83 (4.7)	38 (0.8)	
Glasgow prognostic scale*	0	284 (25.0)	1104 (42.7)	<0.001
	1	609 (53.5)	1265 (48.9)	
	2	245 (21.5)	219 (8.5)	
BCLC stage	0	132 (7.4)	880 (18.3)	<0.001
	A	638 (35.9)	2364 (49.2)	
	B	186 (10.5)	572 (11.9)	
	C	706 (39.8)	924 (19.2)	
	D	113 (6.4)	64 (1.3)	
ALBI grade	I	631 (35.5)	2860 (59.5)	<0.001
	II	966 (54.4)	1802 (37.5)	
	III	178 (10.0)	142 (3.0)	
Portal vein invasion	Vp0	1235 (69.6)	4224 (87.9)	<0.001
	Vp1	175 (9.9)	253 (5.3)	
	Vp2	82 (4.6)	97 (2.0)	
	Vp3	55 (3.1)	68 (1.4)	
	Vp4	228 (12.8)	162 (3.4)	
T stage	1	210 (11.8)	998 (20.8)	<0.001
	2	650 (22.4)	2254 (46.9)	
	3	640 (36.1)	1300 (27.1)	
	4	275 (15.5)	252 (5.2)	
N stage	0	1558 (87.8)	4599 (95.7)	<0.001
	1	217 (12.2)	205 (4.3)	
M stage	0	1602 (90.3)	4672 (97.3)	<0.001
	1	173 (9.7)	132 (2.7)	
AFP (ng/mL)	Median	91	30	<0.001
	Range	1–600000	1-200000	
PIVKA-II (mAU/mL)	Median	243	40	<0.001
	Range	4–75000	2–75000	
Primary treatment	Liver transplantation	60 (3.4)	68 (1.4)	<0.001
	Hepatectomy	285 (16.1)	1584 (33.0)	
	Radiofrequency ablation	225 (12.7)	1093 (22.8)	
	TACE	824 (46.4)	1791 (37.3)	
	Systemic therapy	139 (7.8)	113 (2.4)	
	Radiotherapy	15 (0.8)	17 (0.3)	
	None	227 (12.8)	138 (2.9)	
NLR	Median	2.7	1.5	<0.001
	Range	0.2–47.8	0.1-18.7	
PLR	Median	116.6	74.7	<0.001
	Range	20.3–638.8	2.8–793.7	

Abbreviation: LMR = lymphocyte to monocyte ratio; ECOG = Eastern Cooperative Oncology Group; HBV = hepatitis B virus; HCV = hepatitis C virus; BCLC = Barcelona Clinic Liver Cancer; ALBI = albumin-bilirubin; AFP = alpha-fetoprotein; PIVKA-II = Protein induced by vitamin K absence or

antagonist-**II**; TACE = trans-arterial chemo-embolization; NLR = neutrophil to lymphocyte ratio; PLR = platelet to lymphocyte ratio. Glasgow prognostic scale*: The C-reactive protein (CRP) test was performed selectively in some patients.

Table S5. Correlation Analysis between Neutrophil to Lymphocyte Ratio (NLR) as the Categorical Variable and Other Variables in the Validation Set.

**Variables**		**NLR < 3 (*n* = 1681)**	**NLR ≥ 3 (*n* = 402)**	***p*-Value**
**Age (years)**	Median	59	58	0.244
	Range	22–89	21-86	
Sex	Male	1331 (79.5)	343 (85.3)	0.005
	Female	350 (20.8)	248 (14.7)	
ECOG performance status	0	1627 (98.5)	376 (95.4)	<0.001
	1	24 (1.5)	17 (41.5)	
	2	0 (0.0)	1 (0.3)	
	3	0 (0.0)	0 (0.0)	
	4	1 (0.1)	0 (0.0)	
Cause of hepatitis	HBV	1257 (74.8)	296 (73.6)	0.01
	HCV	159 (9.5)	23 (5.7)	
	HBV/HCV	13 (0.8)	6 (1.5)	
	Alcohol	108 (6.4)	26 (6.5)	
	Unknown	144 (8.6)	51 (12.6)	
Child-Pugh Class	A	1550 (92.2)	305 (75.9)	<0.001
	B	120 (7.1)	79 (19.7)	
	C	11 (0.7)	18 (4.5)	
Hepatic steatosis index	<30	179 (10.9)	56 (14.4)	0.04
	30-36	785 (47.6)	161 (41.4)	
	>36	684 (41.5)	172 (44.2)	
Glasgow prognostic scale*	0	125 (71.0)	31 (31.0)	<0.001
	1	44 (25.0)	36 (36.0)	
	2	7 (4.0)	33 (33.0)	
BCLC stage	0	362 (21.5)	36 (9.0)	<0.001
	A	666 (39.6)	123 (30.6)	
	B	147 (8.7)	28 (7.0)	
	C	496 (29.5)	197 (49.0)	
	D	10 (0.6)	18 (4.5)	
ALBI grade	I	1285 (85.5)	218 (54.2)	<0.001
	II	370 (22.0)	166 (41.3)	
	III	26 (1.5)	18 (4.5)	
Portal vein invasion	Vp0	1244 (74.0)	232 (57.9)	<0.001
	Vp1	291 (17.3)	45 (11.2)	
	Vp2	1 (0.1)	0 (0.0)	
	Vp3	80 (4.8)	56 (14.0)	
	Vp4	65 (3.9)	68 (17.0)	
T stage	1	328 (19.5)	41 (10.2)	<0.001
	2	721 (42.9)	137 (34.2)	
	3	482 (28.7)	138 (34.4)	
	4	150 (8.9)	85 (21.2)	
N stage	0	1602 (95.3)	349 (87.0)	<0.001
	1	79 (4.7)	52 (13.0)	
M stage	0	1655 (98.5)	370 (92.3)	<0.001
	1	26 (1.5)	31 (7.7)	
AFP (ng/mL)	Median	18	60	<0.001
	Range	1–200000	1–200000	
PIVKA-II (mAU/mL)	Median	53	820	<0.001
	Range	6-75000	10-75000	
Primary treatment	Liver transplantation	17 (1.0)	4 (1.0)	<0.001
	Hepatectomy	676 (40.2)	105 (26.1)	
	Radiofrequency ablation	315 (18.7)	35 (8.7)	
	TACE	544 (32.4)	149 (37.1)	
	Systemic therapy	4 (2.5)	34 (8.5)	
	Radiotherapy	18 (1.1)	19 (4.7)	
	None	69 (4.1)	56 (13.9)	
PLR	Median	80.9	139.7	<0.001
	Range	13.0–355.4	34.4–1491.7	
LMR	Median	4.3	2.2	<0.001
	Range	0.8–40.0	0.2–22.0	

Abbreviation: NLR = neutrophil to lymphocyte ratio; ECOG = Eastern Cooperative Oncology Group; HBV = hepatitis B virus; HCV = hepatitis C virus; BCLC = Barcelona Clinic Liver Cancer; ALBI = albumin-bilirubin; AFP = alpha-fetoprotein; PIVKA-II = Protein induced by vitamin K absence or antagonist-**II**; TACE = trans-arterial chemo-embolization; PLR = platelet to lymphocyte ratio; LMR = lymphocyte to monocyte ratio. Glasgow prognostic scale*: The C-reactive protein (CRP) test was performed selectively in some patients.

Table S6. Correlation Analysis between Platelet to Lymphocyte Ratio (PLR) as the Categorical Variable and Other Variables in the Validation Set.

Variables		PLR < 100 (*n* = 1257)	PLR ≥ 100 (*n* = 826)	*p*-Value
**Age (years)**	Median	59	59	0.169
	Range	22–89	21-88	
Sex	Male	993 (79.0)	681 (82.4)	0.055
	Female	264 (21.0)	145 (17.6)	
ECOG performance status	0	1213 (98.3)	790 (97.3)	0.139
	1	20 (1.6)	21 (2.6)	
	2	0 (0.0)	1 (0.1)	
	3	0 (0.0)	0 (0.0)	
	4	1 (0.1)	0 (0.0)	
Cause of hepatitis	HBV	923 (73.4)	630 (76.3)	<0.001
	HCV	138 (11.0)	44 (5.3)	
	HBV/HCV	12 (1.0)	7 (0.8)	
	Alcohol	89 (7.1)	45 (5.4)	
	Unknown	95 (7.6)	100 (12.1)	
Child-Pugh Class	A	1121 (89.2)	734 (88.9)	0.615
	B	121 (9.6)	78 (9.4)	
	C	15 (1.2)	14 (1.7)	
Hepatic steatosis index	<30	133 (10.8)	102 (12.7)	0.17
	30-36	562 (45.7)	384 (47.6)	
	>36	536 (43.5)	320 (39.7)	
Glasgow prognostic scale*	0	90 (62.9)	66 (49.6)	0.007
	1	41 (28.7)	39 (29.3)	
	2	12 (8.4)	28 (21.1)	
BCLC stage	0	292 (23.2)	106 (12.8)	<0.001
	A	494 (39.3)	295 (35.7)	
	B	119 (9.5)	56 (2.7)	
	C	338 (26.9)	355 (43.0)	
	D	14 (1.1)	14 (1.7)	
ALBI grade	I	892 (42.8)	611 (40.7)	0.259
	II	335 (26.7)	201 (24.3)	
	III	30 (2.4)	14 (1.7)	
Portal vein invasion	Vp0	955 (76.0)	521 (63.2)	<0.001
	Vp1	212 (16.9)	124 (15.0)	
	Vp2	0 (0.0)	1 (0.1)	
	Vp3	52 (4.1)	84 (10.2)	
	Vp4	38 (3.0)	95 (11.5)	
T stage	1	277 (22.0)	92 (11.2)	<0.001
	2	525 (41.8)	333 (40.4)	
	3	351 (27.9)	269 (32.6)	
	4	104 (8.3)	131 (15.9)	
N stage	0	529 (88.2)	1423 (96.0)	<0.001
	1	71 (11.8)	60 (4.0)	
M stage	0	564 (94.0)	1462 (98.6)	<0.001
	1	36 (6.0)	21 (1.4)	
AFP (ng/mL)	Median	15	47	<0.001
	Range	1–200000	1–200000	
PIVKA-II (mAU/mL)	Median	43	409	<0.001
	Range	6–75000	10–75000	
Primary treatment	Liver transplantation	20 (1.5)	1 (0.1)	<0.001
	Hepatectomy	449 (35.7)	332 (40.2)	
	Radiofrequency ablation	266 (21.2)	84 (10.2)	
	TACE	425 (33.8)	268 (32.4)	
	Systemic therapy	27 (2.1)	49 (5.9)	
	Radiotherapy	13 (1.0)	24 (2.9)	
	None	57 (4.5)	68 (8.2)	
PLR	Median	1.6	2.6	<0.001
	Range	0.3–15.3	0.5–46.9	
LMR	Median	4.5	3.1	<0.001
	Range	0.7–40.0	0.2–22.0	

Abbreviation: PLR = platelet to lymphocyte ratio; ECOG = Eastern Cooperative Oncology Group; HBV = hepatitis B virus; HCV = hepatitis C virus; BCLC = Barcelona Clinic Liver Cancer; ALBI = albumin-bilirubin; AFP = alpha-fetoprotein; PIVKA-II = Protein induced by vitamin K absence or antagonist-**II**; TACE = trans-arterial chemo-embolization; NLR = neutrophil to lymphocyte ratio; LMR = lymphocyte to monocyte ratio. Glasgow prognostic scale*: The C-reactive protein (CRP) test was performed selectively in some patients.

Table S7. Correlation Analysis between Lymphocyte to Monocyte Ratio (LMR) as the categorical variable and other variables in the validation set.

Variables		LMR < 3 (*n* = 599)	LMR ≥ 3 (*n* = 1482)	*p*-Value
**Age (years)**	Median	59	59	0.306
	Range	22–89	21–88	
Sex	Male	525 (87.5)	1149 (77.5)	<0.001
	Female	75 (12.5)	334 (22.5)	
ECOG performance status	0	567 (96.4)	1436 (98.5)	0.004
	1	20 (3.4)	21 (1.4)	
	2	1 (0.2)	0 (0.0)	
	3	0 (0.0)	0 (0.0)	
	4	0 (0.0)	1 (0.1)	
Cause of hepatitis	HBV	418 (69.7)	1135 (76.5)	<0.001
	HCV	44 (7.3)	138 (9.3)	
	HBV/HCV	5 (0.8)	14 (0.9)	
	Alcohol	61 (10.2)	73 (3.5)	
	Unknown	72 (12.0)	123 (8.3)	
Child-Pugh Class	A	465 (77.5)	1390 (93.7)	<0.001
	B	114 (19.0)	85 (5.7)	
	C	21 (3.5)	8 (0.5)	
Hepatic steatosis index	<30	61 (10.5)	174 (12.0)	<0.001
	30-36	235 (40.4)	711 (48.9)	
	>36	286 (49.1)	570 (39.2)	
Glasgow prognostic scale*	0	108 (77.7)	48 (35.0)	<0.001
	1	25 (18.0)	55 (40.1)	
	2	6 (4.3)	34 (24.8)	
BCLC stage	0	73 (12.2)	325 (21.9)	<0.001
	A	186 (31.0)	603 (40.7)	
	B	50 (8.3)	125 (8.4)	
	C	270 (45.0)	423 (28.5)	
	D	21 (3.5)	7 (0.5)	
ALBI grade	I	303 (50.5)	1200 (80.9)	<0.001
	II	269 (44.8)	267 (18.0)	
	III	28 (4.7)	16 (1.1)	
Portal vein invasion	Vp0	367 (61.3)	1109 (74.8)	<0.001
	Vp1	79 (13.2)	257 (17.3)	
	Vp2	0 (0.0)	1 (0.1)	
	Vp3	71 (11.9)	65 (4.4)	
	Vp4	82 (13.7)	51 (3.4)	
T stage	1	79 (13.2)	290 (19.6)	<0.001
	2	197 (32.9)	661 (44.6)	
	3	206 (34.4)	414 (27.9)	
	4	117 (19.5)	118 (8.0)	
N stage	0	1558 (87.8)	4599 (95.7)	<0.001
	1	217 (12.2)	205 (4.3)	
M stage	0	1602 (90.3)	4672 (97.3)	<0.001
	1	173 (9.7)	132 (2.7)	
AFP (ng/mL)	Median	19	34	<0.001
	Range	1–200000	1–200000	
PIVKA-II (mAU/mL)	Median	53	301	<0.001
	Range	6–75000	8–75000	
Primary treatment	Liver transplantation	11 (1.8)	10 (0.7)	<0.001
	Hepatectomy	135 (22.5)	646 (43.6)	
	Radiofrequency ablation	74 (12.3)	276 (18.6)	
	TACE	238 (39.7)	455 (30.7)	
	Systemic therapy	45 (7.5)	31 (2.1)	
	Radiotherapy	29 (4.8)	8 (0.5)	
	None	68 (3.3)	57 (3.8)	
PLR	Median	1.6	3.1	<0.001
	Range	0.3–7.2	0.4–46.9	
LMR	Median	80.0	121.1	<0.001
	Range	13.0–451.1	25.5–1491.7	

Abbreviation: LMR = lymphocyte to monocyte ratio; ECOG = Eastern Cooperative Oncology Group; HBV = hepatitis B virus; HCV = hepatitis C virus; BCLC = Barcelona Clinic Liver Cancer; ALBI = albumin-bilirubin; AFP = alpha-fetoprotein; PIVKA-II = Protein induced by vitamin K absence or antagonist-**II**; TACE = trans-arterial chemo-embolization; NLR = neutrophil to lymphocyte ratio; PLR = platelet to lymphocyte ratio. Glasgow prognostic scale*: The C-reactive protein (CRP) test was performed selectively in some patients.

Table S8. Reference ranges of blood cell categories associated with systemic inflammation markers.

**Blood cell categories**	**Reference Ranges**
Lymphocytes (absolute count)	1,500–4,000/μL
Neutrophil (absolute count)	1,570–8,300/μL
Platelet (absolute count)	141,000–316,000/μL (male)<br>138,000–347,000/μL (female)
Monocyte (%)	2.2–8.2% of white blood cell count (male)<br>1.7–8.0% of white blood cell count (female)
White blood cell (absolute count)	3,800–10,580/μL (male)<br>3,150–8,630/μL (female)

© 2020 by the authors. Submitted for possible open access publication under the terms and conditions of the Creative Commons Attribution (CC BY) license (http://creativecommons.org/licenses/by/4.0/).
